# Scoliosis in Mitochondrial Myopathy: Case Report and Review of the Literature

**DOI:** 10.1097/MD.0000000000000513

**Published:** 2015-02-13

**Authors:** Zheng Li, Jianxiong Shen, Jinqian Liang

**Affiliations:** From the Department of Orthopaedic Surgery, Peking Union Medical College Hospital, Chinese Academy of Medical Sciences and Peking Union Medical College, Beijing, China.

## Abstract

The mitochondrial myopathies include a diverse group of disorders characterized by morphological abnormalities of muscle mitochondria. Little is reported about spinal deformity associated with this syndrome.

This study presents a case of scoliosis occurring in the setting of mitochondrial myopathies and explores the possible mechanisms between the 2 diseases.

A previously unreported scoliosis in mitochondrial myopathies is described. The patient was a 16-year-old Chinese adolescent boy undergoing a posterior correction at thoracic 2-lumbar 3 (T2-L3) levels using the Moss-SI spinal system. At 48-month follow-up, the patient was clinically pain free and well balanced. Plain radiographs showed solid spine fusion with no loss of deformity correction. After evaluating 60 mitochondrial myopathies, patients referred to Peking Union Medical College Hospital from February 2009 to May 2013, the prevalence of scoliosis among patients with mitochondrial myopathies was 5% (3/60), much higher than that among general population (2%).

The scoliosis in mitochondrial myopathies is usually extensive and progressively aggravated and the correction of the associated spinal deformities is generally difficult. Therefore, the exact role of mitochondrial myopathy in the development of scoliosis requires further study for a better understanding of the disease, as well as adequate and effective patient care.

## INTRODUCTION

Mitochondrial myopathies are a diverse group of disorders characterized by morphological abnormalities of muscle mitochondria.^1^ Mitochondrial disorders are the most common form of inherited metabolic disorders with an incidence of 1 in 4000.^2,3^ Engel et al first reported this disease in 1966 and subsequent cases were reported with strikingly similar findings on histochemical and electron microscopic studies.^4^ Patients with this disorder have a wide spectrum of symptoms due to varied genotype penetrance and disease severity.^2^ Patients may present with fibromyalgia, skeletal muscle weakness, ptosis, pain, fatigue, and exercise intolerance that progressively worsens over time.^5^ In addition, patients may present with slowly progressive peripheral muscle weakness, multisystem organ failure, or respiratory insufficiency requiring mechanical ventilation.^6^ There are no specific therapeutic strategies for mitochondrial myopathies.^7^ The exact cause, pathogenesis, and embryologic origin of mitochondrial myopathies remain a subject of discussion. There are limited reports regarding the diagnosis and management of mitochondrial myopathies with its possible resultant scoliosis. We here present a case in a 16-year-old boy with mitochondrial myopathies and scoliosis and explore the possible association between them.

## CONSENT

Written informed consent was obtained from the patient's parents on behalf of the child for publication of this case report and any accompanying images. A copy of the written consent is available for review by the editor of this journal.

## CASE REPORT

**FIGURE 1:**
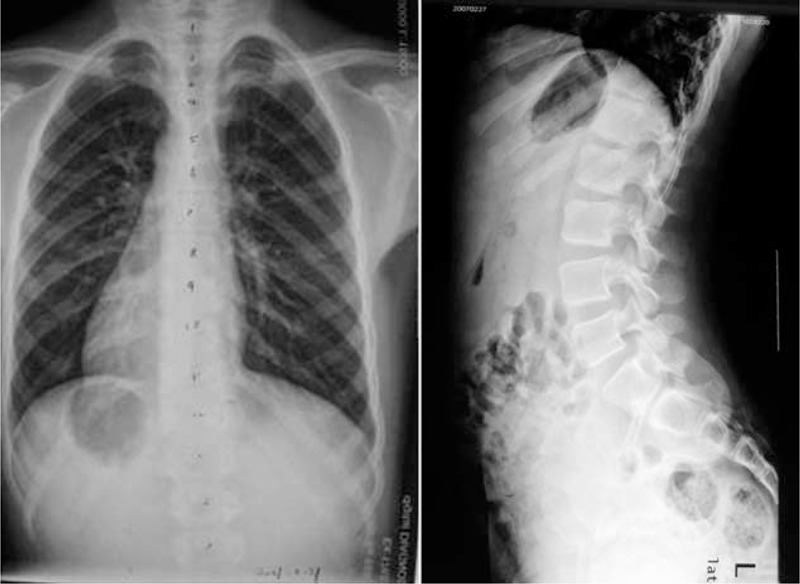
Standing anteroposterior and lateral radiographs of the preoperation on February 2007.

**FIGURE 2:**
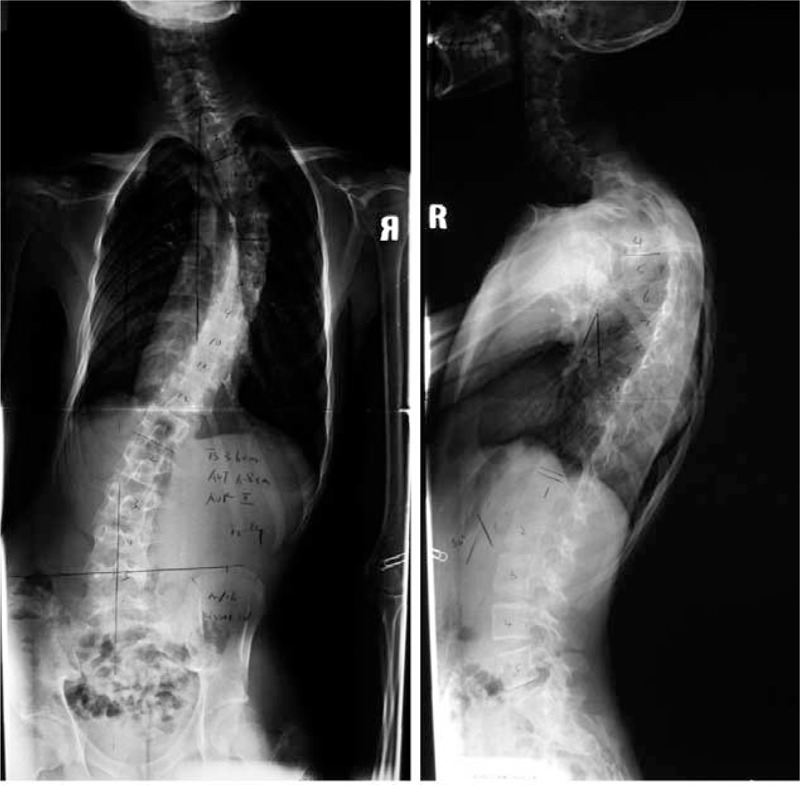
Standing anteroposterior and lateral radiographs of the preoperation on December 2009.

**FIGURE 3:**
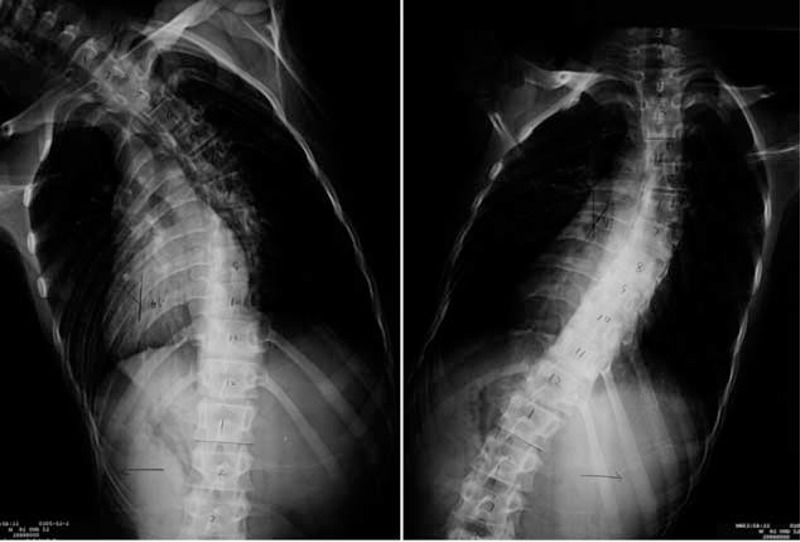
All length right and left side bending x-ray of spine on December 2009.

We present the case of a 16-year-old asylum seeker who was admitted for correction of his progressive scoliosis. His plain radiographs of the spine showed that the thoracic scoliosis was progressive with the Cobb angles increasing from 0° to 53° from February 2007 (Figure 1) to December 2009 (Figures 2 and 3), suggesting the need for surgical correction.

His past medical history was only remarkable in that he had complained about progressive muscle weakness of limbs since the age of 12. He had undergone a skeletal muscle biopsy in the Department of Neurology in our hospital that revealed the typical appearance of mitochondrial myopathies with multiple deletions of mitochondrial DNA. His physical signs included muscle weakness of limbs and tendon reflex decrease. The patient denied any recent dysphagia, pain, and expiratory dyspnea. Magnetic resonance imaging (MRI) revealed no evidence of any spinal cord or canal abnormalities. Computed tomography (CT) revealed no vertebral body deformities. The family history was unremarkable, and there was no parental consanguinity. The diagnosis of mitochondrial myopathies was confirmed based on the findings of skeletal muscle biopsy.

**FIGURE 4:**
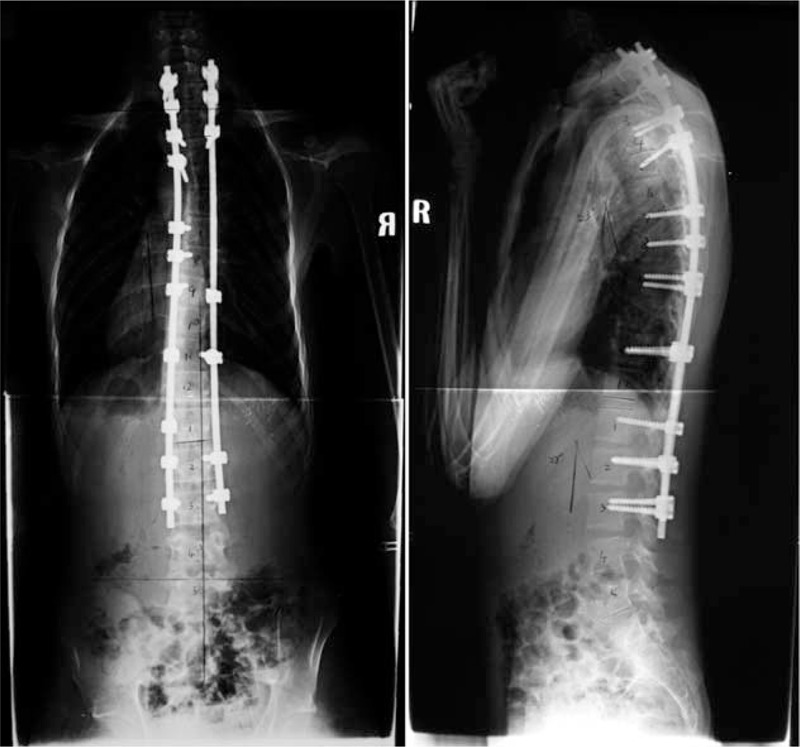
Standing anteroposterior and lateral radiographs of 4 days after operation.

**FIGURE 5:**
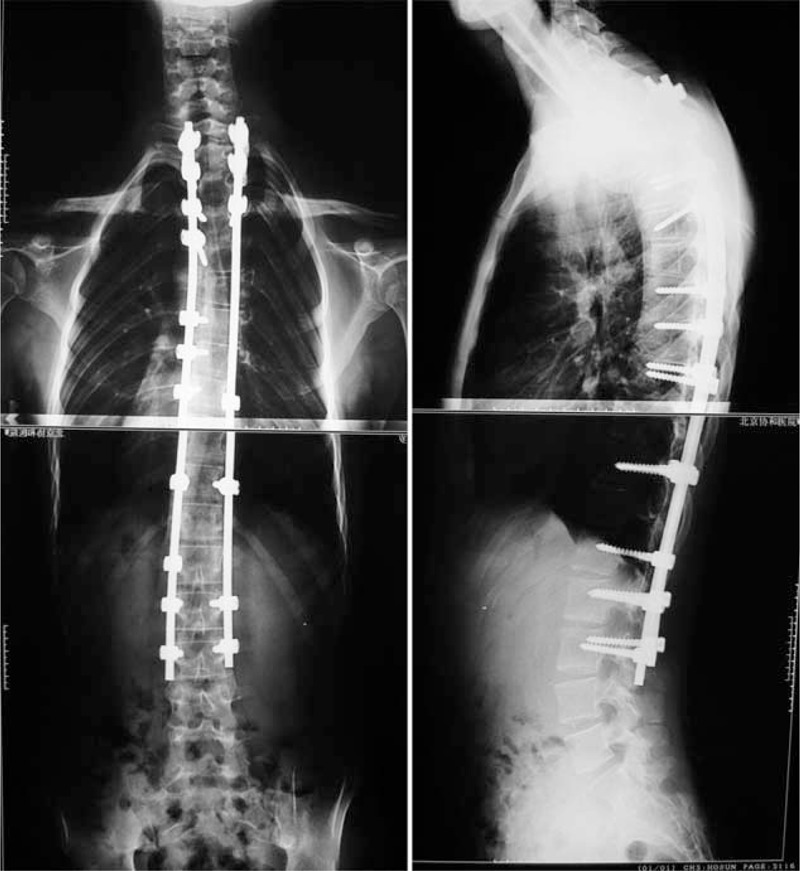
Standing anteroposterior and lateral radiographs of 48 months after operation.

In January 2010, a posterior correction and fusion at T2-L3 levels were performed, using the Moss-SI spinal system (Johnson & Johnson, New Brunswick, American). The total operation time was about 4 hours. Total amount of blood loss was 400 mL. During the operation, the signal of this patient is normal using intraoperative spinal cord monitoring. Postoperatively, there was no sign of respiratory dysfunction. Postoperative plain x-ray film demonstrated a Cobb angles correction from 53° to 8° (correction rate 85%) (Figure 4). He was asymptomatic, well balanced in both the sagittal and coronal planes, with solid fusion at the 48-month postoperative follow-up (Figure 5). Both the patient and families were satisfied with the results of surgery.

To study the potential association between scoliosis and mitochondrial myopathies, we evaluated the spinal deformity of the mitochondrial myopathies patients in our hospital between February 2009 and May 2013. All mitochondrial myopathies were diagnosed based on the clinical examination and muscle biopsy. A total of 60 mitochondrial myopathies patients were identified. Among them, there were 3cases of scoliosis. The prevalence of scoliosis among patients with mitochondrial myopathies was 5%, much higher than the incidence of scoliosis among general population (2%).

## DISCUSSION

The prevalence of mitochondrial myopathies worldwide was estimated to be 0.025%; however, the accurate frequency of mitochondrial disorders is difficult to estimate because of their clinical and genetic heterogeneity.^8^ There are limited reports regarding the diagnosis and management of mitochondrial myopathies with scoliosis. In the present study, we reported a case of a 16-year-old mitochondrial myopathies patient with scoliosis. To our knowledge, this is the first report of scoliosis in the setting of mitochondrial myopathies.

Mitochondria are the only organelles that have their own DNA (mitochondrial DNA or mtDNA), and their own cellular machinery for making RNA and protein.^9^ All mitochondria are maternally inherited; mutations in mtDNA are passed on to all offspring of an affected mother, but only transmitted further to her daughters.^9,10^ Diagnosis of mitochondrial myopathy is often a challenge clinically. Typically, patients may present with slowly progressive peripheral muscle weakness, multisystem organ failure, or solely respiratory insufficiency requiring mechanical ventilation.^11^ Many forms of this disorder manifest in adolescence or childhood and have a very poor prognosis.^12,13^ Electromyographic testing often shows evidence of active or inactive myopathy. Molecular genetic testing and muscle biopsy are usually performed to confirm the diagnosis.^2^ Many characteristic features can be seen on light microscopy, including detection of ragged red fibers on modified trichrome Gomori stain and ragged blue fibers on SDH stain. Absence of COX staining is often seen in mitochondrial myopathy patients.^14,15^ The diagnosis of mitochondrial myopathy was confirmed based on the points of muscle biopsy. To our knowledge, the scoliosis among mitochondrial myopathy has not been reported. There are no specific treatment guidelines on patients of scoliosis with mitochondrial myopathy. Doctors must keep in mind that the respiratory function impairment might progress in these mitochondrial myopathy patients, possibly to abnormal before surgery. During surgery, all the general anesthetic agents are known to directly inhibit mitochondrial function and may add to perioperative problems.

The exact etiology and pathogenesis of mitochondrial myopathy with scoliosis are still not known. The prevalence of scoliosis among patients with mitochondrial myopathy was 5%, much higher than the incidence of scoliosis that among general population (2%).^16^ The clinical performance of this case is similar to the scoliosis with the neuromuscular disorders. Neuromuscular disorders are a group of diseases affecting the neuromusculoskeletal system. Children with neuromuscular disorders frequently develop progressive spinal deformities with cardiorespiratory compromise in the most severe cases.^17^ The main limitations for investigating the association between scoliosis and mitochondrial myopathy are the sample size, since both of them were rare disease.

## CONCLUSION

In conclusion, mitochondrial myopathy is a relatively novel rare syndrome described in recent years. When performing surgery on patients of scoliosis with mitochondrial myopathy, surgeons and anesthesiologists should mind the associated respiratory function impairment. The prevalence of scoliosis among patients with mitochondrial myopathies was much higher than that among general population. There is a potential association between scoliosis and mitochondrial myopathy. However, the exact association of all these conditions is unclear. As the number of cases increases, the etiology, clinical manifestations, and natural history of scoliosis with mitochondrial myopathy will become clearer with more investigations.

## References

[1] Pitceathly RD McFarland R Mitochondrial myopathies in adults and children: management and therapy development. *Curr Opin Neurol* 2014; 27:576–582. 25188013 10.1097/WCO.0000000000000126

[2] Milone M Wong LJ Diagnosis of mitochondrial myopathies. *Mol Genet Metab* 2013; 110:35–41. 23911206 10.1016/j.ymgme.2013.07.007

[3] Hassani A Horvath R Chinnery PF Mitochondrial myopathies: developments in treatment. *Curr Opin Neurol* 2010; 23:459–465. 20651591 10.1097/WCO.0b013e32833d1096

[4] Engel AG Late-onset rod myopathy (a new syndrome?): light and electron microscopic observations in two cases. *Mayo Clin Proc* 1966; 41:713–741. 5957590

[5] Tarnopolsky MA Raha S Mitochondrial myopathies: diagnosis, exercise intolerance, and treatment options. *Med Sci Sports Exerc* 2005; 37:2086–2093. 16331134 10.1249/01.mss.0000177341.89478.06

[6] Taivassalo T Haller RG Exercise and training in mitochondrial myopathies. *Med Sci Sports Exerc* 2005; 37:2094–2101. 16331135 10.1249/01.mss.0000177446.97671.2a

[7] Schoser BG Pongratz D Extraocular mitochondrial myopathies and their differential diagnoses. *Strabismus* 2006; 14:107–113. 16760117 10.1080/09273970600701218

[8] Wisely NA Cook PR General anaesthesia in a man with mitochondrial myopathy undergoing eye surgery. *Eur J Anaesthesiol* 2001; 18:333–335. 11350477 10.1046/j.0265-0215.2001.00852.x

[9] Muravchick S Clinical implications of mitochondrial disease. *Adv Drug Deliv Rev* 2008; 60:1553–1560. 18647627 10.1016/j.addr.2008.03.019

[10] Poulton J Marchington DR Segregation of mitochondrial DNA (mtDNA) in human oocytes and in animal models of mtDNA disease: clinical implications. *Reproduction* 2002; 123:751–755. 12052229 10.1530/rep.0.1230751

[11] Tatke M Mitochondrial myopathies-clinicopathological features and diagnostic modalities. *Indian J Pathol Microbiol* 2007; 50:467–477. 17883111

[12] Temiz P Weihl CC Pestronk A Inflammatory myopathies with mitochondrial pathology and protein aggregates. *J Neurol Sci* 2009; 278:25–29. 19101700 10.1016/j.jns.2008.11.010

[13] Pfeffer G Chinnery PF Diagnosis and treatment of mitochondrial myopathies. *Ann Med* 2013; 45:4–16. 21867371 10.3109/07853890.2011.605389 PMC3581062

[14] DiMauro S Pathogenesis and treatment of mitochondrial myopathies: recent advances. *Acta Myol* 2010; 29:333–338. 21314015 PMC3040593

[15] Sharp LJ Haller RG Metabolic and mitochondrial myopathies. *Neurol Clin* 2014; 32:777–799.ix. 25037090 10.1016/j.ncl.2014.05.001

[16] Longworth B Fary R Hopper D Prevalence and predictors of adolescent idiopathic scoliosis in adolescent ballet dancers. *Arch Phys Med Rehabil* 2014; 95:1725–1730. 24662812 10.1016/j.apmr.2014.02.027

[17] Canavese F Rousset M Le Gledic B Surgical advances in the treatment of neuromuscular scoliosis. *World J Orthop* 2014; 5:124–133. 24829875 10.5312/wjo.v5.i2.124 PMC4017305

